# A Preliminary Concurrent Validation of a Portable Markerless Motion Capture System Against Three-Dimensional Gait Analysis Following Total Hip and Knee Arthroplasty

**DOI:** 10.3390/s26196204

**Published:** 2026-09-30

**Authors:** Mark Abdelnour, Adam Hart, Patrik Abdelnour, Shawn Robbins, Karen Smith, Tanya Capolicchio, Maria Do Carmo Correia De Lima, Michael Tanzer

**Affiliations:** 1 Surgical and Interventional Sciences, Department of Surgery, McGill University, Montreal, QC H3G 1A4, Canada; mark.abdelnour@mail.mcgill.ca (M.A.);; 2 Division of Orthopaedic Surgery, Department of Surgery, McGill University, 1650 Cedar Avenue, Montreal, QC H3G 1A4, Canada; 3 Jo Miller Orthopaedic Research Laboratory, Montreal General Hospital, Montreal, QC H3G 1A4, Canada; 4 School of Physical and Occupational Therapy, Faculty of Medicine and Health Sciences, McGill University, Montreal, QC H3G 1Y5, Canada

**Keywords:** clinical biomechanics, depth sensing, kinematics, spatiotemporal outcomes

## Abstract

Three-dimensional gait analysis (3DGA) in patients undergoing total hip and knee arthroplasty (THA and TKA, respectively) is impractical for widespread clinical use. This study evaluated the Azure Kinect, combined with the Motognosis AMSA software (AK-M), for the assessment of selected clinically relevant gait parameters in THA and TKA patients compared with reference 3DGA. Twenty participants (10 THA, 10 TKA) were simultaneously assessed using AK-M and Qualisys 3DGA while walking along an 8 m walkway. Discrete peaks of sixteen bilateral gait parameters were compared using Bland–Altman mean bias and 95% limits of agreement (LOAs), with ICC(3,1) as a complementary measure of relative consistency. Maximum hip kinematics and knee extension demonstrated low mean bias. In contrast, maximum knee flexion, knee range of motion, and step length demonstrated larger mean bias and wider LOAs. Cadence and gait speed demonstrated the lowest bias and narrowest LOAs. ICC(3,1) values ranged from 0.84 to 0.95. AK-M showed parameter-specific agreement with 3DGA, supporting preliminary concurrent validity for simplified assessment of selected discrete gait outcomes. Wider LOAs for other parameters identify areas requiring further technical refinement and clinical interpretation. These findings apply to the combined arthroplasty cohort and should not be interpreted as separate THA and TKA cohorts.

## 1. Introduction

Gait analysis has been growing over the years across various clinical populations, especially in total hip arthroplasty (THA) and total knee arthroplasty (TKA) patients [1]. This objective assessment of patients’ movement patterns and functional recovery is useful in both clinical and research settings [1,2]. Clinically, the application of gait analysis includes identifying abnormal walking patterns, guiding operative and non-operative management, and monitoring recovery following surgery [2,3,4,5]. From a research perspective, gait analysis allows for understanding movement patterns in various clinical populations, assessing the effectiveness of surgical procedures or rehabilitation programs over time, and investigating outcome prediction [1,2,5]. As the applications of gait analysis expand, the use of reliable and accessible gait analysis tools becomes increasingly important.

Currently, the gold standard measurement tool for gait analysis is optoelectronic motion analysis systems [6]. Such systems typically include several high-speed infrared digital cameras placed around a specific area designated for the performance of a task, with a floor instrumented with a force plate [6]. Reflective markers attached to participants over anatomic landmarks allow high-speed digital cameras to recognize specific anatomical landmarks [6]. Together, the three-dimensional motion analysis system setup allows accurate and reliable measurement of spatiotemporal, kinematic and kinetic gait parameters [7].

Although traditional gait analysis labs provide measurements of high reproducibility and accuracy [8], they also require an elaborate setup with specialized equipment that is extremely costly, facilities that are not readily accessible to physicians, and technologists [9,10]. In addition, a complete analysis of the aforementioned parameters is time-consuming and the results may be difficult for practitioners without a biomechanical background to interpret, which are well established factors that hinder the use of standardized outcome measures [11,12]. Therefore, a portable, low-cost, and simple motion capture device capable of quantifying selected clinically relevant gait parameters would facilitate the integration of gait analysis in the routine evaluation of THA and TKA patients.

One such simple motion capture system is the Azure Kinect (AK; Microsoft Corporation, Redmond, WA, USA) combined with the Motognosis AMSA software (Version 1.2, Motognosis, GmbH, Berlin, Germany), referred to as AK-M. The AK-M has been used for gait analysis in clinical populations, including patients with Parkinson’s disease, cerebral palsy, and stroke [13,14,15]. In THA and TKA, other portable technologies, including smartphone-based measurements, wearable inertial sensors, and markerless assessment of functional tasks, have been investigated [16,17,18]. However, these approaches have generally assessed a limited number of parameters, required wearable sensors, or focused on specific functional tasks. AK-M is an established markerless system capable of simultaneously assessing multiple bilateral kinematic and spatiotemporal gait parameters during walking. To our knowledge, concurrent comparison of such a system with laboratory-based 3DGA in patients following THA and TKA remains limited.

The purpose of this prospective, cross-sectional preliminary concurrent validity study was to evaluate agreement between the Azure Kinect combined with Motognosis AMSA software (AK-M) and the Qualisys (AB, Gothenburg, Sweden) three-dimensional gait analysis system (3DGA) for selected discrete kinematic and spatiotemporal gait parameters in patients following total hip arthroplasty (THA) and total knee arthroplasty (TKA).

## 2. Materials and Methods

### 2.1. Study Design

This was a prospective, cross-sectional preliminary concurrent validity study evaluating agreement between AK-M and the Qualisys 3DGA system for selected discrete gait parameters (Miqus M3, Qualisys, Sweden). The study was conducted at a gait analysis laboratory. This study was approved by the Institutional Review Board (IRB Study Number MP-37-2022-8613). In-person written informed consent was obtained from all the participants. In order to protect confidentiality, all data were coded with participant ID numbers, securely stored, and accessible only to the research team.

### 2.2. Participants

Participants were recruited from the Orthopedic Surgery Clinic from January 2022 to August 2024. Inclusion criteria consisted of individuals who underwent a primary unilateral or bilateral THA or TKA within the last 2 years, and aged 18–85 years at the time of surgery. Exclusion criteria included participants with a revision arthroplasty or complex primary arthroplasty with a bone graft, arthritis in their other knee or hip joints, or other conditions that impact the participant’s ability to ambulate, such as lower extremity trauma requiring surgery within the past year of enrollment, inflammatory arthritis, and severe neurological impairments. Twenty participants (10 THA and 10 TKA) were included in this study (Table 1), which is consistent with previous validation studies of motion capture systems [19,20,21,22].

### 2.3. Experimental Setup

**Figure 1:**
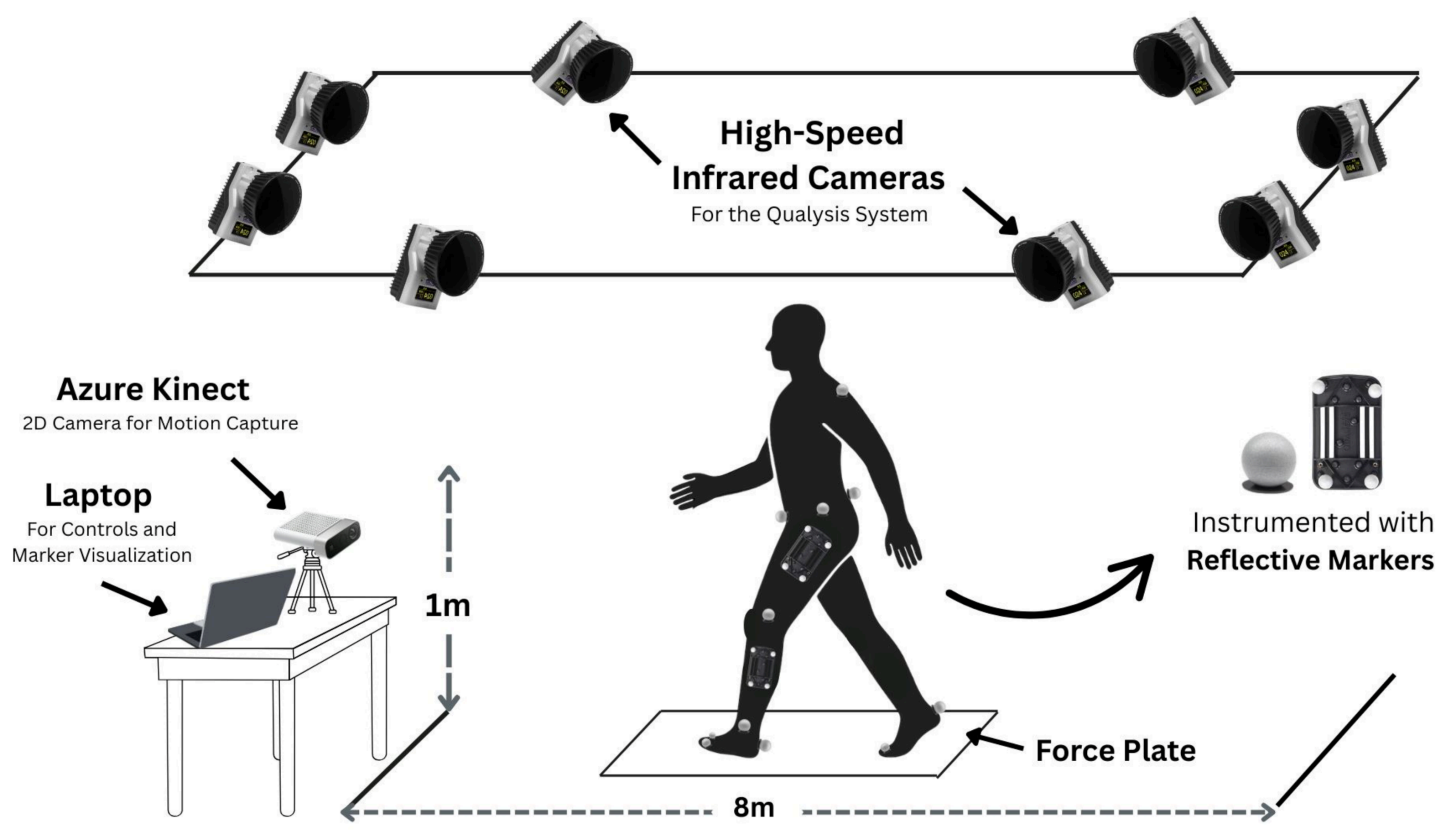
Graphic illustration of experimental setup during walking trials.

All participants wore tight-fitting shorts and were barefoot to facilitate visualization of the lower extremity joint. Forty reflective markers were positioned according to the six-degrees-of-freedom marker set described by Collins et al. [23] Anatomical markers were placed at the anterior superior iliac spines (ASIS), posterior superior iliac spines (PSIS), greater trochanters, medial and lateral knee condyles, medial and lateral malleoli, calcanei, and the first, second, and fifth metatarsal heads, with rigid marker clusters on the lateral thighs and shanks. Following the static calibration trial, markers at the bilateral greater trochanters, medial knee condyles, and medial malleoli were removed for dynamic trials. The gait analysis recorded the participant walking back and forth down an 8 m walkway at a comfortable pace, as shown in Figure 1. The participants were allowed two practice trials before the recorded gait study and were not asked to target the force plates. When necessary, the starting position was adjusted, and trials were repeated to obtain clean force-plate contacts while preserving a self-selected walking pattern. Testing proceeded until 5 successful trials were collected. The Azure Kinect camera was positioned 4 m anterior to the force plates so that the force-plate contact occurred within the Kinect capture window, ensuring that the same stride was analyzed by both systems.

#### 2.3.1. Reference 3D Motion Capture System

Data were collected using the eight-optical-camera Qualisys 3D motion capture system sampled at 100 Hz. Data collection began with a static trial and a standing template was constructed to ensure the lower extremity rotation was consistent [23]. The static trial was used to determine joint centers and as an anatomic calibration. The floor was instrumented with two force plates, sampled at 2000 Hz, which served to identify gait events such as heel strike and toe-off. Only strides that included force plate strike were analyzed.

#### 2.3.2. AK-M Markerless Motion Capture System

**Figure 2:**
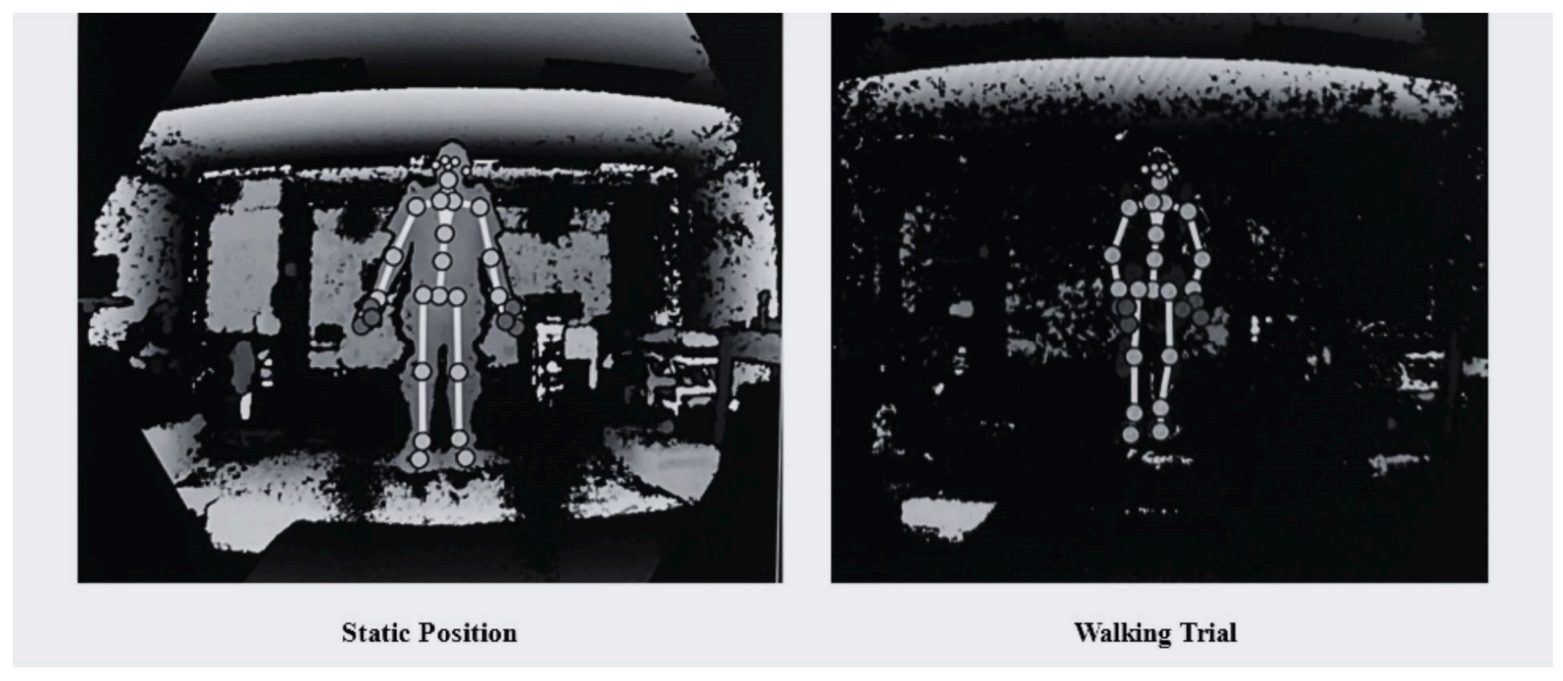
Images of the outputs from the Azure Kinect displayed on the Motognosis AMSA software during the static position and walking trial.

Data was collected using the Azure Kinect sampled at 30 Hz simultaneously with the Qualisys system. This markerless system uses an RGB-Depth sensor that emits a grid of infrared light. The distance of objects within the camera’s recording range was used to compute a depth model of surrounding structures [24]. A motor-task-specific kinematic software (Motognosis AMSA software, Version 1.2; Motognosis, Berlin, Germany) was then used to create depth silhouettes of individuals, in which 25 different anatomical landmarks are located [24], as shown in Figure 2. The Azure Kinect camera was positioned at the end of the walking plane, facing the participant during walking, at a height of 1 m and 0° inclination. The RGB-D data were processed by the Motognosis AMSA software to generate the anatomical landmark and joint-kinematic outputs used in the analysis.

### 2.4. Data Analysis

#### 2.4.1. Data Processing

All data processing for Qualisys and AK-M trials was conducted with commercial software Visual3D (v5; C-motion Inc., Germantown, MD, USA) and Motognosis AMSA software (Motognosis, Berlin, Germany), respectively.

Preprocessing of Qualisys data was done to correct for missing values. A low-pass Butterworth filter (8 Hz for marker data, 20 Hz for force plate data) was applied to reduce noise while preserving meaningful movement and force signals. Ankle and knee joint centers were defined as the midpoints between the medial and lateral malleoli and femoral epicondyles, respectively; hip joint centers were estimated using published methods [25,26]. Knee joint angles were calculated in Visual3D using a joint coordinate system approach (Grood–Suntay), with flexion defined as positive [27]. Heel strike and toe-off were identified from the vertical ground-reaction force to define the gait cycle. The AK-M data were analyzed directly as captured using the Motognosis AMSA software, with no additional preprocessing steps. As a part of the standard AK-M machine learning processing software, large jumps in the movement data (due to occlusions or errors in tracking) were detected and excluded from the analysis, together with the derived ROM and both corresponding Qualisys values from that trial.

The 16 gait parameters analyzed included bilateral maximum hip flexion, maximum hip extension, and hip range of motion (ROM); bilateral maximum knee flexion, maximum knee extension, and knee ROM; bilateral mean step length; cadence; and gait speed. Step length was defined as the distance covered between consecutive foot contacts of opposite limbs, cadence as the number of steps per minute, and gait speed as the distance traveled per unit time. For Qualisys, gait events were identified using vertical ground-reaction force data from the force plates. AK-M spatiotemporal parameters were generated directly by the Motognosis AMSA software using its existing proprietary processing pipeline. Joint angles were reported with flexion positive and extension negative. Maximum hip and knee extension represented the most extended joint position during stance and corresponded to the lowest joint-angle value, whereas maximum hip and knee flexion corresponded to the highest joint-angle value during swing. ROM was calculated within each trial as the difference between the highest and lowest joint-angle values. Spatiotemporal outcomes were analyzed at the stride level. These phase-specific discrete outcomes were selected because they represent clinically relevant peak joint positions [28,29,30].

#### 2.4.2. Statistical Analyses

Gait parameters extracted from successful trials were averaged for each participant prior to analysis. Right- and left-sided outcomes were then analyzed separately, with each participant contributing one value per side. Absolute agreement between AK-M and Qualisys was assessed using Bland–Altman analysis, reporting mean bias (the average difference between measurements from the two systems) and 95% limits of agreement (LOA = mean bias ± 1.96 × SD of the paired differences) [31,32]. Mean bias was defined as Qualisys − AK-M, such that a positive bias indicates lower values measured by AK-M relative to Qualisys. The standard deviation (SD) of the paired differences was used to describe the random component of between-system disagreement. For clinical interpretability, Bland–Altman analysis was interpreted in the original units (degrees or cm). Ninety-five percent confidence intervals (CIs) were also calculated for the mean bias and each limit of agreement to describe the precision of the Bland–Altman estimates. As a complementary measure of relative consistency between systems, intraclass correlation coefficients (ICC [3,1]) were calculated using a two-way mixed-effects, single-measure model. ICC values greater than 0.75 were interpreted as indicating excellent consistency in biomechanical studies [33]. All analyses were performed in STATA 17.2 (StataCorp, College Station, TX, USA).

## 3. Results

The mean and SD for each gait parameter measured by the AK-M and Qualisys systems are presented in Table 2.

**Figure 3:**
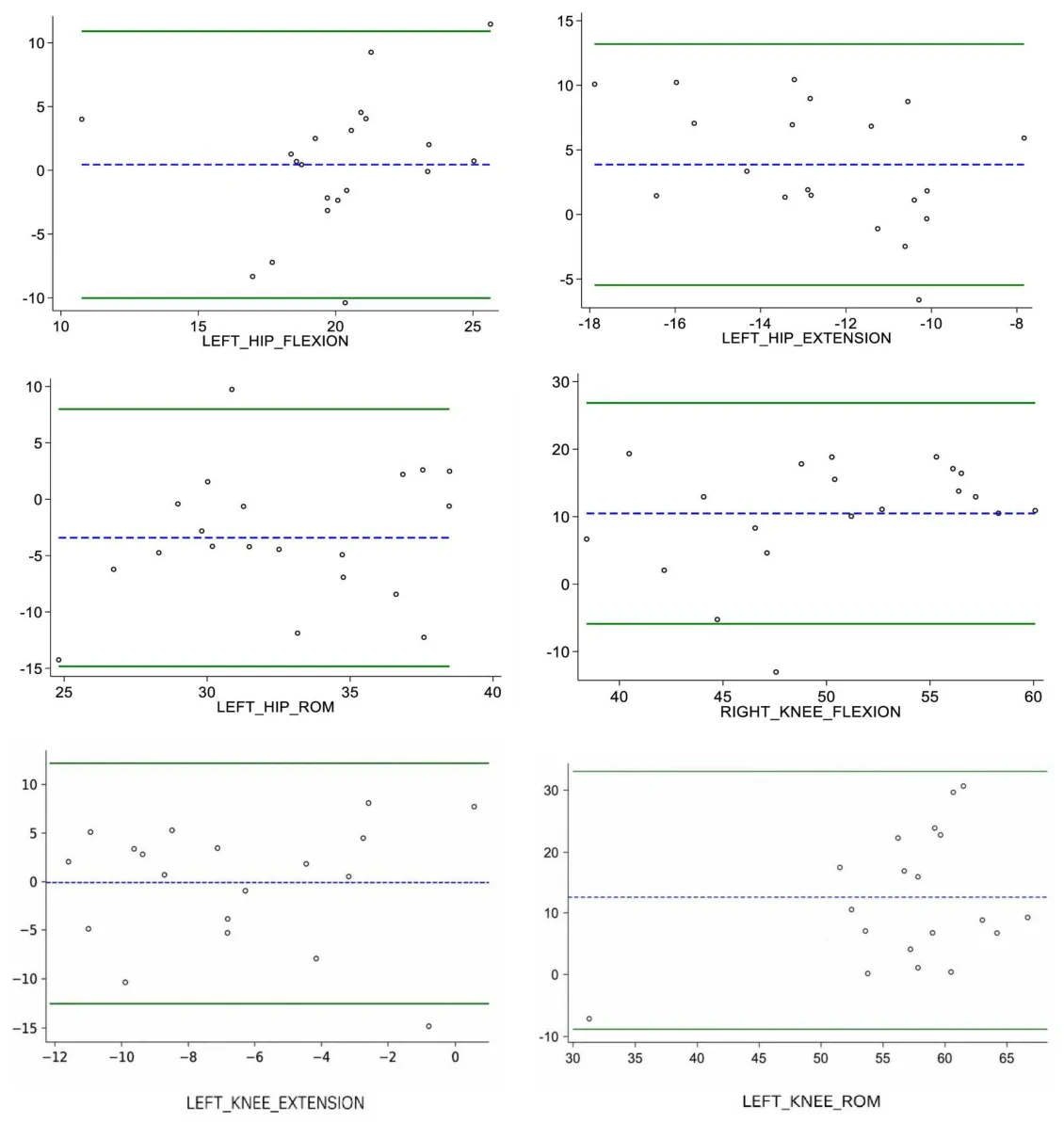
Bland–Altman plots comparing AK-M and Qualisys for bilateral hip and knee kinematic parameters. The y-axis represents the paired difference (Qualisys − AK-M), and the x-axis represents the mean of the two systems. The dashed blue line represents the mean bias, and the solid green lines represent the 95% limits of agreement.

**Figure 4:**
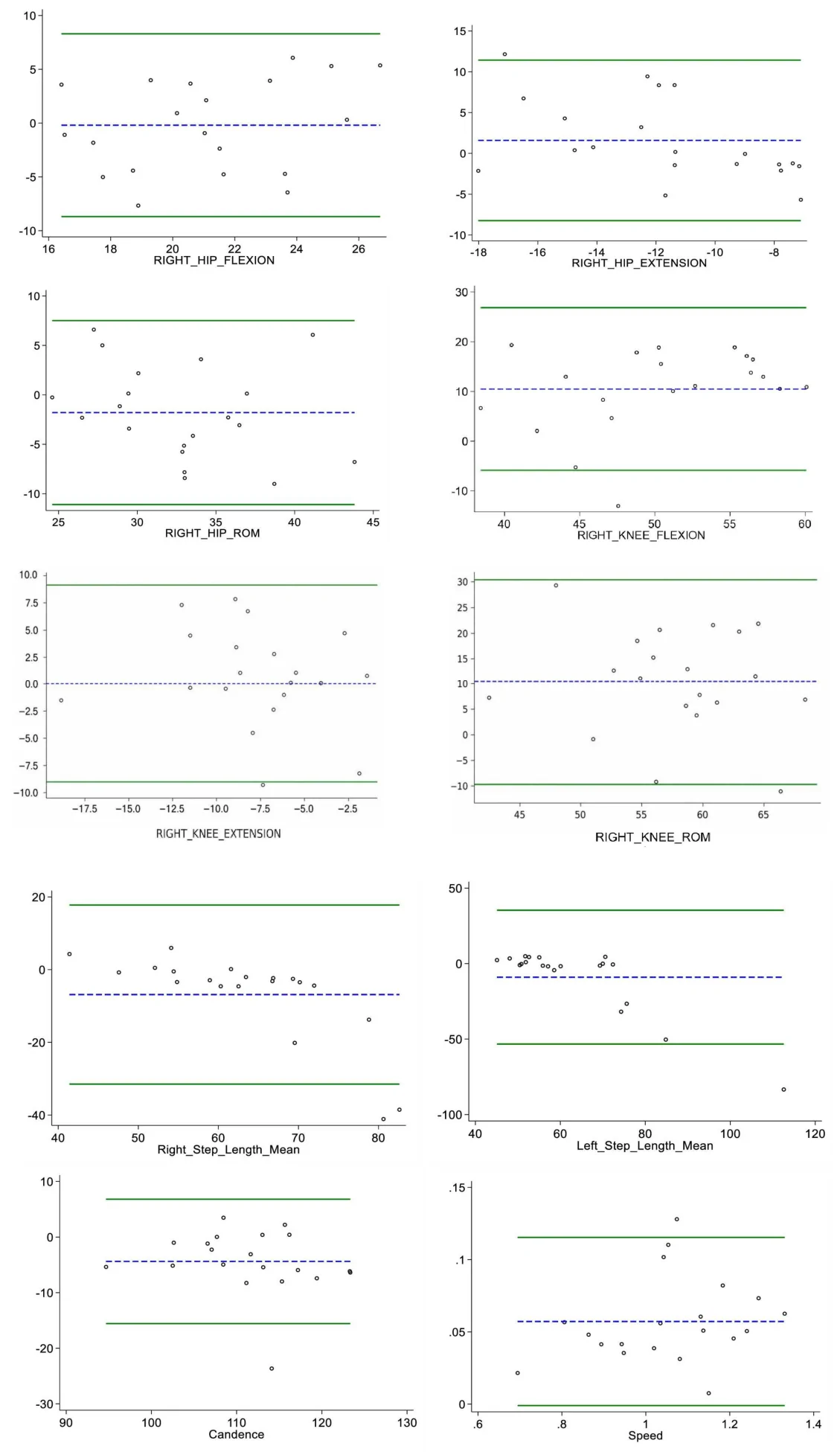
Bland–Altman plots comparing AK − M and Qualisys for hip and knee spatiotemporal parameters. The y-axis represents the paired difference (Qualisys − AK-M), and the x-axis represents the mean of the two systems. The dashed blue line represents the mean bias, and the solid green lines represent the 95% limits of agreement.

Bland–Altman analysis including mean bias, SD of paired differences, and 95% limits of agreement (LOAs) are summarized in Table 3. Bland–Altman plots for kinematic and spatiotemporal parameters are presented in Figure 3 and Figure 4, respectively.

Hip kinematics demonstrated low systematic differences between AK-M and Qualisys, with mean biases close to zero to a few degrees and low-to-moderate variability. For hip flexion, mean bias was close to zero (right: −0.20°, left: 0.44°), with SD of differences of 4.30–5.34° and LOAs spanning approximately −10° to +11°. Hip extension showed slightly larger positive biases (right: 1.60°, left: 3.86°), indicating AK-M tended to report more negative extension values than Qualisys, with LOAs of −8.23° to 11.42° (right) and −5.47° to 13.19° (left). Hip ROM biases were low and negative (right: −1.79°, left: −3.42°), with LOA widths ranging from approximately 19° to 23°, indicating notable individual-level spread despite low average bias.

Knee kinematic agreement was parameter-dependent. For knee extension, mean bias was close to zero (right: 0.20°, left: −0.30°), with SD of differences of 4.69–6.20° and LOAs of −8.99° to 9.40° (right) and −12.45° to 11.85° (left), indicating low systematic differences with low-to-moderate variability. In contrast, knee flexion demonstrated larger systematic differences (mean bias right: 10.48°, left: 12.05°), with SD of differences of 8.35–9.57° and LOAs of −5.89° to 26.80° (right) and −6.70° to 30.82° (left). This indicates that the AK-M measured lower knee flexion values compared with Qualisys, with higher variability of the paired differences. A similar pattern was observed for knee ROM where mean bias was positive (right: 10.37°, left: 11.88°), with SD of differences of approximately 10° and LOAs of −9.59° to 30.34° (right) and −8.88° to 32.64° (left).

Spatiotemporal agreement was parameter-dependent. Step length demonstrated wide limits of agreement, particularly on the left side. Mean bias was negative (right: −6.85 cm, left: −8.99 cm), with SD of differences of 12.59 cm (right) and 22.61 cm (left), and LOAs of −31.49 to 17.78 cm (right) and −53.31 to 35.33 cm (left), indicating high variability and limited absolute agreement.

Cadence demonstrated a mean bias of −4.38 steps/min, with an SD of differences of 5.71 steps/min and LOAs of −15.57 to 6.81 steps/min, indicating low systematic bias with low-to-moderate variability. Gait speed demonstrated a low mean bias of 0.06 m/s with narrow LOAs (−0.001 to 0.11 m/s), indicating the closest absolute agreement among the spatiotemporal outcomes.

ICC(3,1) values ranged from 0.84 to 0.95, demonstrating strong relative consistency between AK-M and Qualisys across the evaluated gait parameters (all *p* < 0.001), see Table 4.

## 4. Discussion

This preliminary concurrent validity study evaluated agreement between AK-M and the reference Qualisys 3DGA system for selected clinically relevant gait parameters to determine its potential for simplified outpatient gait assessment in patients following THA and TKA. Overall, our results demonstrated that agreement between AK-M and Qualisys was parameter-dependent. Maximum hip flexion and extension, maximum knee extension, cadence, and gait speed showed low systematic differences, whereas maximum knee flexion, knee ROM, and step length showed larger systematic offsets and wider LOAs, suggesting that clinical utility may differ across parameters. These findings provide preliminary evidence that a portable markerless system can capture selected clinically relevant gait outcomes in an arthroplasty population, while also identifying parameters that require greater caution and further technical refinement.

The observed agreement for hip kinematics is consistent with prior validation studies of Azure Kinect-based systems, which have reported more favorable performance for proximal joint angles compared with distal measures [22,34,35]. Proximal segments such as the pelvis and thigh are generally tracked with greater robustness in RGB-D pose estimation, which likely contributes to the relatively stable agreement observed for hip flexion and extension. From a clinical perspective, this is relevant, as hip kinematics are commonly used to characterize functional recovery and compensatory strategies after THA and TKA [36,37]. The ability to quantify these patterns using a portable, low-cost system may therefore have potential for simplified outpatient gait assessment.

In contrast, the agreement of discrete peak knee kinematics was dependent on the specific parameter examined. Measures related to knee extension demonstrated low systematic differences and more consistent agreement, suggesting that knee extension may be a promising parameter for further evaluation with AK-M. Both systems recorded mean maximum knee-extension angles of approximately −6° to −8°, indicating extension beyond the model-defined neutral position. Because knee hyperextension was not assessed clinically, it is unclear whether these values represent true hyperextension, differences in the model-defined neutral position, or both. Concerning parameters derived from knee flexion and ROM, larger systematic differences and greater variability were demonstrated. Similar findings have been reported in previous Kinect-based validation studies, where knee flexion-related measures showed lower agreement than hip kinematics [22,24,38]. These discrepancies indicate that although AK-M captures general knee motion patterns, knee flexion and knee ROM derived from AK-M lack the absolute agreement required for direct comparison with 3DGA and are therefore unsuitable for precise clinical or biomechanical decision-making.

Low agreement for knee flexion likely reflects known limitations of distal lower-limb tracking in single-sensor RGB-D systems. Prior studies show that ankle and foot landmarks exhibit higher noise and lower signal-to-noise ratios than proximal joints, which can propagate into shank and tibial segment definitions and affect knee angle estimation [22,24]. Furthermore, single-camera configurations are sensitive to viewing angle, depth-axis motion, and transient self-occlusion during gait, disproportionately impacting distal segments [19,39]. Finally, the lower sampling frequency of AK-M (30 Hz) compared with Qualisys (100 Hz) may also contribute to underestimation of short-duration peak values, particularly maximum knee flexion. These well-established limitations may explain the observed parameter-specific differences without implying system-specific deficiencies.

Spatiotemporal parameters similarly demonstrated variable agreement. Gait speed and cadence showed comparatively low systematic differences, consistent with previous reports indicating that spatiotemporal metrics are among the most robust outputs of Azure Kinect-based gait analysis systems [40,41]. In contrast, step length demonstrated poorer agreement, which aligns with prior findings that step-based spatial measures are particularly sensitive to inaccuracies in distal landmark localization and gait event detection [38,42]. Given that step length is derived from foot position and timing, even small errors in distal tracking can lead to substantial between-system variability, which may limit the accuracy of step length estimates.

Whether agreement is “sufficient” depends on intended use [31]. For THA and TKA, gait measures are most commonly used for screening rather than degree-level biomechanical decision-making. As a priori clinical acceptability thresholds were not defined in this preliminary study, the observed agreement should be interpreted in a parameter-specific manner rather than as evidence of interchangeability with 3DGA. In the present study, hip kinematics, knee extension, cadence, and gait speed demonstrated lower systematic bias and variability and may therefore be more informative for simplified clinical gait assessment, whereas knee flexion, knee ROM, and step length demonstrated greater disagreement and warrant greater caution. This parameter-specific contrast provides a clinically relevant framework for interpreting AK-M outputs according to their intended use.

The complementary ICC(3,1) analysis demonstrated strong relative consistency between AK-M and Qualisys across the evaluated gait parameters, with ICC values ranging from 0.84 to 0.95. This indicates that participants with relatively higher or lower gait measurements on Qualisys tended to show similarly higher or lower measurements on AK-M, supporting the ability of AK-M to consistently capture between-participant differences. These findings are consistent with previous Kinect-based studies reporting strong relative agreement for gait and functional movement measures [24,35,41]. Taken together, the ICC findings complement rather than replace the Bland–Altman analysis, as strong relative consistency does not necessarily imply close absolute agreement between systems.

There were several limitations to this study. First, the relatively small sample size may limit generalizability to all patients undergoing THA or TKA. Second, because this study focused exclusively on arthroplasty patients, the findings may not be transferable to other orthopedic or neurological populations. Third, data collection was performed using a single-sensor configuration in a controlled outpatient setting; agreement may differ with alternative camera placement or in less controlled clinical environments, particularly for parameters dependent on distal lower-limb tracking. Fourthly, formal assessment of Bland–Altman assumptions, including proportional bias and heteroscedasticity, was not performed; therefore, the possibility that disagreement may vary across the measurement range cannot be excluded. The frequency of tracking failures and excluded trials could not be reliably determined retrospectively; therefore, the technical failure rate and clinical feasibility of AK-M could not be fully assessed. The present study did not assess within-session repeatability, test–retest reliability, minimal detectable change, or responsiveness; therefore, conclusions regarding longitudinal measurement performance cannot be made. Finally, THA and TKA participants were analyzed as one mixed arthroplasty cohort. The small subgroup sizes did not permit meaningful arthroplasty-specific analyses, and agreement may differ according to the replaced joint and associated gait pattern. The findings therefore apply to the combined cohort and should not be interpreted as separate validation in THA and TKA populations.

## 5. Conclusions

This preliminary concurrent validity study was intended as an initial proof-of-concept evaluation of the potential role of simplified gait analysis in routine orthopedic practice. Agreement with 3DGA was parameter-dependent, with low systematic differences for maximum hip flexion and extension, maximum knee extension, gait speed, and cadence, and larger systematic offsets and wider LOAs for maximum knee flexion, knee ROM, and step length. These findings support the preliminary, parameter-specific concurrent validity of AK-M, and its potential as a practical, low-cost approach to simplified objective gait assessment in outpatient arthroplasty care, while parameters with wider LOAs require further technical refinement and validation. Finally, these findings apply to the combined arthroplasty cohort and should not be interpreted as separate THA and TKA cohorts.

## Figures and Tables

**Table 1:** Demographic of the study participants (*N* = 20).

Characteristic	THA (*n* = 10)	TKA (*n* = 10)
Age (mean ± SD; years)	66 ± 9	65 ± 8
BMI (mean ± SD; kg/m^2^)	27.6 ± 5.0	28.3 ± 4.1
Sex (*n*; male/female)	8/2	6/4
Surgical Laterality (*n*; unilateral/bilateral)	9/1	7/3
Charnley Classification (*n*, A, B, C)	9 A, 1 B	7 A, 3 B

SD—standard deviation; BMI—body mass index.

**Table 2:** Summary of gait parameter measures from the AK-M and Qualisys systems (*N* = 20).

Gait Parameter	AK-M		Qualisys	
	Mean	SD	Mean	SD
Right Hip Flexion	21.36	3.78	21.09	4.34
Right Hip Extension	−12.33	5.46	−10.61	3.07
Right Hip ROM	33.69	6.64	31.70	4.98
Right Knee Flexion	44.96	6.98	55.88	8.54
Right Knee Extension	−7.85	4.97	−7.65	4.2
Right Knee ROM	52.67	8.64	63.05	7.5
Left Hip Flexion	19.99	3.83	20.34	4.80
Left Hip Extension	−14.19	4.49	−10.58	2.69
Left Hip ROM	34.18	5.24	30.92	5.45
Left Knee Flexion	43.71	7.04	56.33	8.87
Left Knee Extension	−6.40	4.78	−6.70	4.79
Left Knee ROM	50.97	7.11	62.85	10.67
Right Step Length Mean	68.22	24.21	60.62	7.19
Left Step Length Mean	65.55	34.73	59.12	7.92
Cadence	113.77	12.11	109.72	6.97
Speed	1.04	0.16	1.10	0.16

ROM—range of motion. Joint angles were reported with flexion positive and extension negative.

**Table 3:** Summary of Bland–Altman analysis for gait parameters comparing AK-M and Qualisys systems.

Gait Parameter	Mean Bias	95% CIs of Bias	SD Paired Differences	95% Limits of Agreement (LOAs)	95% CI Lower LOAs	95% CI Upper LOAs
Right Hip Flexion (°)	−0.2	(−2.21, 1.81)	4.3	(−8.70, 8.30)	(−12.20, −5.20)	(4.80, 11.80)
Right Hip Extension (°)	1.6	(−0.76, 3.96)	5.04	(−8.23, 11.42)	(−12.33, −4.13)	(7.32, 15.52)
Right Hip ROM (°)	−1.79	(−4.01, 0.43)	4.75	(−11.09, 7.51)	(−14.95, −7.23)	(3.65, 11.37)
Right Knee Flexion (°)	10.48	(6.57, 14.39)	8.35	(−5.89, 26.80)	(−12.68, 0.90)	(20.01, 33.59)
Right Knee Extension (°)	0.20	(−1.99, 2.39)	4.69	(−8.99, 9.40)	(−12.81, −5.17)	(5.58, 13.22)
Right Knee ROM (°)	10.37	(5.60, 15.14)	10.19	(−9.59, 30.34)	(−17.88, −1.30)	(22.05, 38.63)
Left Hip Flexion (°)	0.44	(−2.06, 2.94)	5.34	(−10.02, 10.90)	(−14.36, −5.68)	(6.56, 15.24)
Left Hip Extension (°)	3.86	(1.63, 6.09)	4.76	(−5.47, 13.19)	(−9.34, −1.60)	(9.32, 17.06)
Left Hip ROM (°)	−3.42	(−6.14, −0.70)	5.82	(−14.82, 7.99)	(−19.56, −10.08)	(3.25, 12.73)
Left Knee Flexion (°)	12.05	(7.57, 16.53)	9.57	(−6.70, 30.82)	(−14.49, 1.09)	(23.03, 38.61)
Left Knee Extension (°)	−0.3	(−3.20, 2.60)	6.20	(−12.45, 11.85)	(−17.49, −7.41)	(6.81, 16.89)
Left Knee ROM (°)	11.88	(6.92, 16.84)	10.59	(−8.88, 32.64)	(−17.50, −0.26)	(24.02, 41.26)
Right Step Length (cm)	−6.85	(−12.74, −0.96)	12.59	(−31.49, 17.78)	(−41.73, −21.25)	(7.54, 28.02)
Left Step Length (cm)	−8.99	(−19.57, 1.59)	22.61	(−53.31, 35.33)	(−71.70, −34.92)	(16.94, 53.72)
Cadence (steps/min)	−4.38	(−7.05, −1.71)	5.71	(−15.57, 6.81)	(−20.22, −10.92)	(2.16, 11.46)
Speed (m/s)	0.06	(0.05, 0.07)	0.03	(−0.001, 0.11)	(−0.03, 0.02)	(0.09, 0.13)

SD = standard deviation; ROM = range of motion. Joint angles were reported with flexion positive and extension negative.

**Table 4:** Intra-class correlations (3,1) between AK-M and Qualisys gait measurements.

Measurement	ICC(3,1)	95% CI		*p*-Value
Right Hip Flexion	0.89	0.84	0.93	<0.001
Right Hip Extension	0.87	0.81	0.91	<0.001
Right Hip ROM	0.91	0.87	0.94	<0.001
Right Knee Flexion	0.93	0.9	0.96	<0.001
Right Knee Extension	0.85	0.78	0.9	<0.001
Right Knee ROM	0.92	0.88	0.95	<0.001
Left Hip Flexion	0.88	0.83	0.92	<0.001
Left Hip Extension	0.86	0.8	0.91	<0.001
Left Hip ROM	0.90	0.85	0.93	<0.001
Left Knee Flexion	0.92	0.88	0.95	<0.001
Left Knee Extension	0.84	0.77	0.89	<0.001
Left Knee ROM	0.91	0.87	0.94	<0.001
Right Step Length Mean	0.95	0.92	0.97	<0.001
Left Step Length Mean	0.94	0.91	0.96	<0.001
Cadence	0.91	0.87	0.94	<0.001
Speed	0.95	0.92	0.97	<0.001

CI—confidence interval; ROM—range of motion.

## Data Availability

The data presented in this study are available from the corresponding author upon reasonable request. The data are not publicly available because of participant confidentiality and institutional ethics requirements.
